# Information communication and technology in sports: a meticulous review

**DOI:** 10.3389/fspor.2023.1199333

**Published:** 2023-07-03

**Authors:** Nahida Reyaz, Gulfam Ahamad, Mohd Naseem, Javed Ali, Khalid Imam Rahmani

**Affiliations:** ^1^ Department of Computer Sciences, Baba Ghulam Shah Badshah University, Rajouri, India; ^2^ College of Computing and Informatics, Saudi Electronic University, Riyadh, Saudi Arabia

**Keywords:** sports, cricket, information and communication technology (ICT), convolutional neural networks (CNN), hawkeye, computer vision, artificial intelligence

## Abstract

**Introduction:**

Sports of all kinds even though have an alluring property of keeping their onlookers stuck to their place, the introduction of Technology, however, revolutionized it all together. Not only in legal sports but also the training and teaching methods have been reformed. The use of Information Communication and Technology (ICT) based technologies [Convolutional Neural Networks (CNN), Hawkeye, Computer vision, Artificial intelligence, etc.] has moderately increased the interactive nature of sports. Employing ICT-driven technologies have continuously been increasing performance levels due to which high effective performance levels have been achieved. In addition to offering information to the users, it also acts as a means for connecting and interacting with the remaining world. In this article, we provide a review of the studies considering the developments and impact of employing ICT technology on sports, especially cricket. The study has focussed on domain-specific developments in cricket sports: developments in the batting domain, bowling domain, and wicketkeeping as well.

**Methods:**

For the study, the analysis has been done following the PRISMA guidelines.

**Results:**

The study found that even though the researchers have done justifiable work in employing technology in sports as a whole but the domain-specific contribution in sports like cricket is not at the level as is need of the hour. In addition to the mentioned domains in the study, the research should gain speed in other domains like domain-specific Talent Identification for both genders, different age groups, diverse sports, etc.

**Discussion:**

undoubtedly, the sports domain is employing technology at a vast level but a few domains like sports talent identification especially related to the most famous games like cricket require an immediate and intense focus of the researchers. Since this domain is still carrying out a traditional coach-oriented approach. There is an acute need to revolutionize the domain by incorporating modern technologies into it.

## 1. Introduction

The introduction of technology into sports has transformed sporting goods, instructional materials, and biological technology (1) thus revolutionizing the sports domain. The phrase “information and communications technology” (ICT) refers to the expansive technological instruments and facilities used only to generate and disperse, store, and manage knowledge (2). The hasty expansion of technologies, having a diverse impact on society as a whole is becoming more and more apparent, and we find ourselves in a constant state of transition and improvement. The modern world is driven by information and communication technology (ICT), influencing engineering, Sports, management, health, tourism, economics, and communication. All are related because either ICT is a tool or using ICT to solve problems is a common practice (3). In addition to the application of modern ICT technologies like Artificial Neural networks in social media (4, 5). ICT expands the digital environment and enhances accessibility in the field of physical education and sport (6). It allows users to link up with and communicate about the globe as a whole in addition to allowing them to gain knowledge, it transforms and modifies the way physical education and sports are taught (7). ICT is very helpful in sports for a variety of reasons, including the fact that video officials use ICT to help them see things that would be difficult to observe otherwise. The outcomes are more precise and well-organized thanks to the usage of ICT. Additionally, ICT offers proof to the authorities so they won't face objections or criticism from the opponent, such as when a tennis ball is marked as out. We can consider the “hawk eye” as a simple example (8). Utilizing information technology, the bar for sports can be raised by: updating the players' health state, scheduling the training, taking regular feedback from the player, practice videos, evaluating physical activity with the use of technology, archiving the outcomes, and evaluating the group's performance during the tournament. Validating profile and online signups. Through information technology, the Coaches can evaluate themselves, retain athletes' personal information, and develop a search engine for the most recent sports training (9). ICT has an ample impact on Talent identification in the sports domain also. In many athletic events, it's critical to identify prospective future elite performers. Early detection of prospective top athletes would make it easier to equip them with high-calibre coaching and training environments that would maximize their growth. However, to achieve in professional sports, a wide range of unique skills and attributes are required, making talent identification a generally complex and diverse challenge. Elite athletes are uncommon, so datasets are naturally unbalanced, which makes conventional statistical inference challenging. One such study approaches the challenge of talent identification as one of the anomaly detection problems. To identify prospective future elite players, a one-class support vector machine (non-linear) was trained on a set of data having 951 entries of Young Soccer players 14 years of age (10).

About a century ago, England is where cricket first appeared (11). Cricket is played with a bat and ball, with each team with 11 players in the case of test cricket players play for 5 days, for 1 day of cricket there are 50 overs played, or more than 4 h (Twenty20) (12). To dismiss the batsman, the bowler launches from one end of the 22-yard line with a straight arm, hoping to hit the wicket at the other end, sending the ball flying into the fielder's reach, or causing one of a variety of other mistakes. The batsman tries to protect the set of three stumps with the bat and runs are scored, the game's currency, by striking the ball close to the field boundary or sufficiently far away from the catchers so that he or she can rush to the opposite side of the field even before the ball could be handed back. There must be at least two bowlers who take turns. It is a one-on-one fight between a single bowler and a single batter, assisted by a team of fielders (13). Around 2.5 billion individuals of all age groups and abilities participate in the popular sport of cricket (14). In India, over 5 million (15), 1.4 million in Australia (16), and close to 300,000 in the UK play cricket (17). Youth also like playing cricket (18), and many of them do so well into adulthood (19). With women accounting for more than 27% of all Australian cricketers, women are also becoming more and more interested in the game (16).

**Figure 1:**
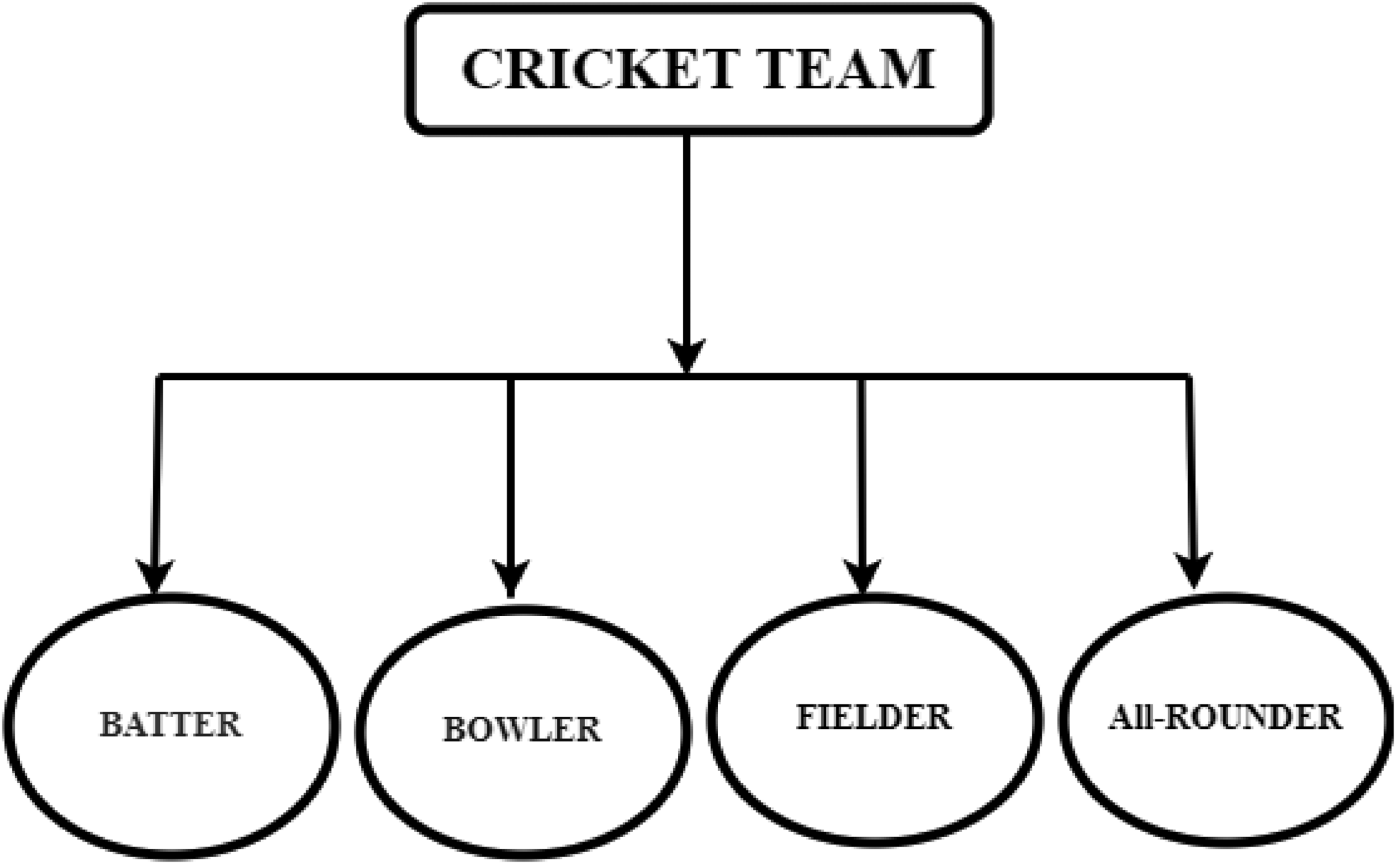
Categories of players in cricket teams based on roles played by them.

As discussed above since cricket is a bat-and-ball type of game, the action starts as only two members of the batting team may be on the field whilst the game is being played; however, all the 11 team members of the fielding team need to be present. Bowler is a player of the fielding squad; the rest nine players are called fielders. The wicketkeeper is the fielder who is positioned behind the wicket to catch any balls that might miss the wicket while the bowler attempts to hit them with the ball. The batter from the opposing team will try to strike the bowled ball before it hits the wicket. When the batsman strikes the ball, the remaining fielders must go after it. The batsman's job is to bat the ball away to stop it from hitting the wicket. Additionally, after a ball is struck, both batsmen must run as many times as they can from their respective wickets to the other, to score a run that is known as the “currency” of cricket (20). Now since there are eleven cricketers on a team. These cricketers typically play the four roles of wicketkeeper, bowler, all-rounder, and batter as depicted in Figure 1.

ICT has been incorporated in all these roles; we came across an ample amount of literature that fairly justifies that ICT has not impacted sports only but almost all types of sports including cricket incredibly as well.

The aim of this article is to perform a review study on the research carried out in the domain of developing the relationship between the sports and ICT by incorporating the technologies emerging everyday into the different sports aspects. The study reviewed the articles mainly concerned to the Cricket sport and witnessed the fact of how beautifully the ICT has impacted the different aspects of the cricket sport.

The research is a novel study as we found no such study from literature that has provided such a detailed review of the works done in the different domains of any sport like done by the study for one of the most famous sports like cricket.

We are going to represent our study in 5 sections. Section 1 aims to provide an elaborate introduction to understanding the relationship between sports and ICT. Section 2 describes the methodology used to review the studies. In Section 3 the review of literature is revealed in tabular form, which makes it appropriate for comparing the results. In Section 4, we unveil and highlight the key findings. Section 5 concludes (wraps up the article) and provides perspective on potential possible improvements.

## 2. Methodology

**Figure 2:**
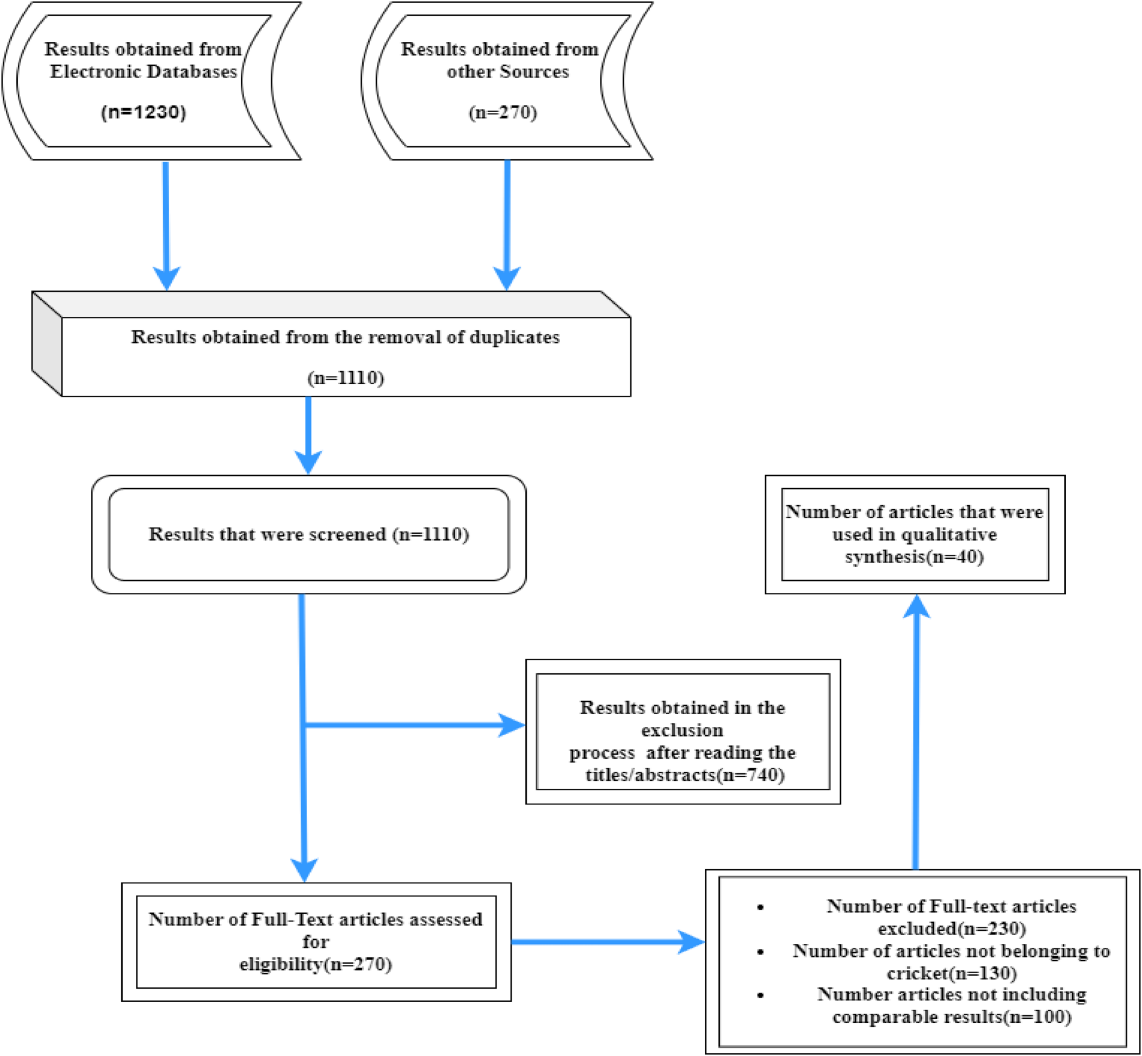
Screening process of articles for review.

The methodology for exploring the literature on the impact of ICT on Sports especially the different domains of cricket is depicted by Figure 2: a pictorial representation of the overall methodology followed for the Review Process.

The Preferred Reporting Items for Systematic Reviews and Meta-Analysis were used in the current review (PRISMA) to assess the articles. The study conducted a targeted search, to sum up almost every aspect that has been impacted by ICT in sports, especially in cricket sport.

### 2.1. Selection criteria for review studies

#### 2.1.1. ICT in sports

The study reviewed a few articles to get an idea and understanding of the impact that ICT has created in sports like in the other aspects of life, e.g., Medicine, Engineering, etc.

#### 2.1.2. ICT in cricket

The study included ample literature witnessing the impact of ICT on the Cricket sport as a whole without taking into consideration the type of technology whether computer vision, machine learning, or any other.

### 2.2. Domain-specific literature

The study tried to get a clear understanding and distinction of how ICT has contributed to different domains of cricket like how it has impacted the Batting domain, bowling domain, fielding domain, and wicketkeeping.

For identifying relevant articles, the search process was carried out by searching from electronic databases like Google scholar, sports discus, web of science, etc. (2008–2022) using PRISMA guidelines. Search strings used were “ICT in Sports”, Impact of ICT on Cricket”, and “Impact of ICT on Bowling, Batting, etc”. The use of relative search strings was done to broaden the search result set. From other relative sources searching was done as a form of secondary search of external sources like the references of the papers obtained in phase 1, additional website searches, and references from books following PRISMA guidelines.

After obtaining articles from the search process the articles were scanned for duplicity in terms of the Study's author(s), title, and year of publication the found duplicities were eliminated. Digging a little deep into the obtained refined list required obtaining and assessing the Full-text articles and Abstracts. Figure 2 depicts the Screening procedure of articles in the review.

## 3. Literature review

The detailed literature survey on how ICT continues to impact all the mentioned domains is:

### 3.1. Use of ICT in batting domain

Batting in cricket means the practice or skill of hitting the ball with a bat used to score runs while keeping one's wicket undamaged. Not necessarily if batting is their area of specialization, any player who is now in the batting position is called a batsman or batswoman, or increasingly, a batter (21). Top-level batters will have quick reflexes, strong diagnoses, and fantastic policy wonks as well as possessing outstanding physical batting talents because they need to adjust to varied situations during playing on different cricket pitches, particularly in diverse countries (22).

The study by Haseeb Ahmad et al. (23) uses machine learning approaches to find up-and-coming cricket players. Two models from each category are tested using generative and discriminative machine learning methods for classification purposes. Study shows rising star prediction with high accuracy that is both reliable and statistically significant using cross-validation. As per the study by Muhammad Zia ul Haq et al. (24). The concerned research compared the pull shot techniques of under-19, under-16, and senior cricket batsmen to quantify coaching descriptions. To compare the biomechanics of the pull shot between groups, a one-way ANOVA with repeated measures was used. As per the study, senior batsmen extended their right knee and left hip much more than batsmen under 16 and batsmen under 19. The left hip's linear velocity was much higher in the under-16 batsmen than it was in the seniors, and the left elbow was higher in the under-19 hitters. The study Muhammad Zeeshan Khan et al. (25) looks at several batting shots from cricket films and classifies them. Deep convolution neural networks are used, and it highlights the significant indications of contemporary AI and deep learning for both identifying different cricket activities and for decision-making. Both 2D convolution and recurrent networks were used to analyse a sequence of video frames, while 3D convolution networks were used to concurrently capture spatial and temporal data 90% of the time, obtained models can accurately identify a shot being played. The study by Chetan Kapadiya et al. (26) suggests a model for predicting the Players' effective performance in the cricket game. The presented model refines the entire team's effectiveness helping in the best team selection. Cricket statistics, as well as weather-related data datasets, are utilized by the model. The model employs a weighted random forest classifier with optimization. The accuracy of the predicted model is found to be good as compared to other algorithms. To categorize batting shots of 10 types from offline footage the research by Anik Sen et al. (27) 2021 suggests a mixed deep-neural network design. Various transfer-learning models that freeze all the layers—namely, VGG16, InceptionV3, Xception, and DenseNet169—were examined. Results of the experiment showed that the VGG16-GRU model performed better than outperformed the other models with an accuracy of 86%. Based on a case study and real-world IoT implementation, the study by Varad Vishwarupe et al. (28) sought to identify a fresh approach to research in cricket analytics referred to as the timing index. The timing index is dependent on a variety of variables, including impact bat speed, maximum bat speed, back lift angle, and bat speed. Such a study is thought to be able to analyse a player's practice sessions utilizing IoT and data analytics, accurately and comprehensively analysing a player's performance, and going beyond conventional statistical reporting by introducing fascinating and illuminating information.

### 3.2. Use of ICT in bowling domain

In the case of cricket, a performer (bowler) throws a ball in the direction of the stumps which another player batsman is ready to protect, this type of action performed is called bowling. The main goals of a bowler are either taking wickets (the act of dismissing batsmen by hitting the stumps with the ball) or preventing the possibility of scoring runs. Bowlers perform deliveries, which are called “bowls,” in sets of six, which make up an “over.” As one bowler finishes an over, a new bowler is brought in to bowl the next set of six balls from the opposite end of the pitch (29).

A neural network technique utilizing a back-propagation algorithm network (BPN) and its radial basis function network (RBFN) has been explored by Shanthi Muthuswamy et al. (30) to estimate the effectiveness of the bowlers on the Indian cricket team. Prediction models performed better for runs than for wickets. The two network paradigms mentioned were used to create a classification strategy for the wickets case. For runs as compared to wickets, prediction models were more successful. The study by Carl Petersen et al. (31) aimed at the quantification of the similar male fast bowler playing One Day International (ODI) cricket matches, time-motion characteristics and intra-athlete diversity in motion patterns were studied. For monitoring several different time-motion characteristics GPS (Global Positioning System) portable 5-Hz unit (Catapult, Melbourne, Australia) was used. It was found that a combination of high-intensity, intermittent activities on a 16-kilometer basis created how this fast bowler moves. This study by Dibyojyoti Bhattacharjee et al. (32) makes an effort to evaluate the effectiveness of a data set of bowlers who participated in season 4 of the Indian Premier League using the combined bowling rate. The reasons that are empirically accountable for the bowlers' performance were then determined using multiple linear regression techniques. Variation in ball speed has a negligible impact on how well bowler performs. Corresponding the variance ratio between the regression equation and the residuals is given as 3.394 with a *p*-value of 0.015. Social network analysis (SNA) is used in the study by Satyam Mukherjee et al. (33) to analyse the effectiveness of a team's players. The study creates a weighted and directed network of batsmen-bowlers using player-vs-player data from Test and ODI cricket. Our findings indicate that M. Muralitharan is the most productive bowler in cricket history. The research purpose Hemanta Saikia et al. (34) is to use an artificial neural network to examine and forecast how bowlers will perform in the IPL. Based on how they performed in the league's first three seasons, the paper attempts to forecast the results of bowlers who decided to join the league in its season 4 as their initial IPL venture. Player real performance in IPL-IV is used to assess the model's external validity. This research M. N. A. Islam et al. (35) suggests a CNN model that uses transfer learning to categorize 18 various cricket bowlers based on their bowling style movements. For training the suggested framework and assessing its effectiveness, a brand-new dataset of 8,100 photographs of these 18 bowlers was also produced. For the model, the study started with the VGG16 model that had already been trained using the ImageNet dataset. The model's test set precision is 93.3%, demonstrating its classification performance. The purpose of this study by Amrinder Singh et al. (36) was to investigate the outcome of different bowling surfaces (natural turf and concrete) on the bowling speed of 41 asymptomatic fast and medium-pacer bowlers who had not sustained any injuries in the 3 months before they participated in the study, particularly to the shoulder joint or back. The statistics indicate a decrease in pitch tempo, which is the difference in pitch speed between the two ends of the pitch, on a pitch with a concrete foundation. In this paper by Rahman R et al. (37), a unique method for determining the delivery style from a bowler's finger grip during delivery was proposed. The primary goal of this research is to accurately classify bowlers' grips using the transfer learning models and the prototype CNN architecture. To train with the GRIP DATASET and analyze grip results, as well as the pre-trained transfer learning models Vgg16, Vgg19, ResNet101, ResNet52, Dense Net, Mobile Net, Alex Net, Inception V3, and Nanette—were used. It was tested for precision, recall, and f1-score, with a maximum average accuracy of 98.6% achieved across 13 classes (13 different bowling actions).

### 3.3. Use of ICT in wicket keeping

In the case of cricket, the wicket-keeper is a player who stands behind the wicket or stumps on the fielding side, keeping an eye on the batter and ready to take a catch, stump the batter, or run out a batter as needed (38). Up to eight out of ten dismissals in one inning in some games involved the wicketkeeper. There is, however, a dearth of writing that is specifically about wicket-keeping. Currently, available information on wicket-keeping is mostly restricted to coaching literature, much of it is based on anecdotal data and recommendations from previous wicket-keepers and instructors (39).

The study Petersen et al. (40) quantified the time-motion characteristics of five cricket positions (Batters, Fast bowlers, Position players, Spin bowlers, and Wicketkeepers) that competed in four State Twenty20 (T20) cricket games. The study used portable 5 Hz global positioning system (GPS) units. The study found that the physical demands of Fielders and Fast bowlers are substantially greater than wicketkeepers and spin bowlers. The study by Dani MacDonald et al. (41) aimed to find out the skill demands and movement in the one-day international match by the use of video analysis. Using the video analysis program Sports Code, television footage from eight games (totalling sixteen innings) of the 2011 one-day international World Cup was examined. The study's findings can help practitioners better grasp the wicket-keeper position's assessment, program, and skill development components.

### 3.4. Developments in cricket as a whole

The study by Subramanian RamaIyer et al. (42) employs neural networks to forecast how well each cricketer will perform in the future based on their previous performance. Cricket players are classified as either performers, middling performers, or failures. The neural network models were gradually trained and tested using four sets of data. The trained neural network models were then used to estimate the cricketer's relatively close performance. The model has a 77% accuracy rate for batting performance and a 63% accuracy rate for bowling performance. The study by Petersen et al. (40) quantified the time-motion characteristics of five cricket positions (Batsmen, Fast bowlers, Fielders, Spin bowlers, and Wicketkeepers) in four State Twenty20 (T20) cricket matches. The study used portable 5 Hz global positioning system (GPS) units. The study found that the physical demands of Fielders and Fast bowlers are substantially greater than wicketkeepers and spin bowlers. To forecast future match events that would result in a win or lose, research by Vignesh Veppur Sankaranarayanan, et al. (43) developed a prediction system that takes into account both data from previous matches as well as the degree of development of a match. Using a selection of match parameters and a hybrid of nearest-neighbour clustering and linear regression, the study models the game. Quantitative findings showed that one of the key factors affecting match outcome is how well our algorithms forecast the number of runs scored. The study by A Z M Ehtesham Chowdhury et al. (44) aimed at concretizing the decision regarding the occurrence or non-occurrence of the no ball. The study made two divisions of the bowling crease, the transformation in pixels then was calculated using the picture decrement technique on two areas. The study eradicated the inadequate nature of human perception as it is based on a pixel-by-pixel image subtraction. The study by Neeraj Pathak et al. (45) aims to forecast the outcome of a One Day International (ODI) cricket match. The match's outcome is influenced by several variables, many of which change as the game progresses, including home field advantage, Day/Night, Toss, Innings (first or second), physical fitness of sides, and dynamic plans. The study compares the results and performances of three contemporary classification techniques—Naive Bayesian, Support Vector Machines, and Random Forest. COP (Cricket Outcome Predictor), a tool that predicts the outcome of an ODI match, was developed about the outcomes of these models. Average balanced accuracy of models: Random Forest 0.6002, SVM 0.6167, and Nave Bayesian 0. 6018. This article by Madan Gopal et al. (46) uses supervised learning to attempt to forecast the outcome of a One Day International (ODI) cricket match based on team composition. The research reveals that the relative team strength of the competing teams is a distinguishing feature for determining the winner. The player was modelled in the study using both his current performances and career statistics. Player-independent elements have also been considered to forecast the outcome of a game. Based on the statistics of 366 matches, the study's accuracy was 71%. The study by Aftab Khan et al. (47) offers a framework for the automated recognition of cricket shots that is both affordable and practical. The movements of the batsmen are captured using body-worn inertial measurement units, and the data is subsequently analysed using a parallelized, hierarchical recognition system that automatically identifies pertinent categories of strokes as necessary for evaluating batting quality. The technology produces accurate visual representations of important performance indicators, such as foot placement, attack/defence, and shot distribution on the playing surface. These visualizations serve as the foundation for an objective skill assessment, concentrating on particular areas for individual growth that the system has identified. F1-score greater than 88%. The study by Pushkar Shukla et al. (48) suggests a model that can create sports highlights automatically, with a concentration on cricket. This research proposes a strategy to identify and clip significant occurrences, the system imbrutes the varied actions taken during a cricket match, allowing the umpire and coach to make accurate decisions during the game. excitement-based attributes. Examples of Replays, audio intensity, player celebration, and playfield scenarios are all used to record such events. CNN + SVM.72.31% is the average precision. To detect umpire poses in the game of cricket, the study Aravind Raviet al. (49) proposes a novel dataset named SNOW. The proposed dataset is assessed as a first step in creating cricket highlights-generating software. According to the study, four such events—SIX, NO BALL, OUT, and WIDE—can be classified based on the umpire's pose as seen in cricket video frames. As the top contenders for feature extraction before training convolutional neural networks such as the Inception V3 and VGG19 networks are used. Player testing accuracy for VGG19-Fc2 was 78.21%. The study by Aman Bhalla et al. (50) suggests for cricket matches a unique strategy for instantly summarising and designed to detect significant events. The input to the model is the entire cricket match in a form of a video recorded, and the most important clips from the game are the output. Many approaches have been used such as sound detection, optical character recognition, and replay detection to excerpt crucial events like wickets, boundaries, and other playfield schemes. The model achieved an 89.45% accuracy in detecting events like wickets, fours, and sixes indicating the significance of the designed technique. The study by Md. Kowsher et al. (51) presents a classification method Convolutional Neural Network (CNN) with Inception-V3 so that decisions of the third umpire and scoring system can be automatically unravelled like signal detection. SoftMax has been applied to find out the likelihood of a match referee judgment and match referee signal classification. To train CNN, pre-formed-V3. was used. The efficacy and effectiveness of the model are found to be high as compared to other methods and applications for detecting no-balls. A 13-layered CNN known as “Shot-Net” is suggested by Md. Ferdouse Ahmed Foysa et al. (52) in the study to classify six different categories of cricket shots played viz. Pull Shot, Scoop Shot, Cut Shot, Cover Drive, Straight Drive, and Leg Glance Shot. The study has focussed on employing Deep Neural Networks as being very useful in different sports data analysing tasks. The accuracy achieved by the model is fairly high and also entropy is low. In this study by Rohit Kumar et al. (53), cricket films were used for the outcome categorization job. The creation of automatic commentary generation is the major goal of such actions. For this assignment, there are a lot of sub-tasks that must be taken into account. Classifying the result of each ball for which commentary is to be produced is one of those duties. This paper covers the entire categorization process, from gathering the data to producing the findings. Using Long Short-Term Memory with Convolutional Neural Networks in the game of cricket, there are four main outcomes: Run, Dot, Boundary, and Wicket. Cricket match ball-by-ball videos' results have been predicted with an accuracy of 70%. The study by Daniel Mago Vistro et al. (54) aims at predicting the IPL match winner before the game starts. To predict the winner of the IPL machine learning models is trained on the designated features. Different advanced machine learning algorithms are put in for the reason of model building on various test and training datasets like Logistic Regression, Random Forest, SVM, Decision Tree, and Naïve Bayes. For evaluating the team's strength and cricket analysis the model is found to perform well. The study by Hannah K Jowitt et al. (55) for detecting bowling deliveries accurately, automatically, and reliably aimed to devise and check the logic based on a machine learning approach. Inertial sensor data from a Catapult OptimEye S5 wearable device was collected from national as well as global grade fast bowlers (*n* = 35) in both practice and competition at various intensities. A machine-learning-based algorithm comes out to be a reliable tool for detecting bowling events instantaneously, moreover, enabling us to look at performance metrics related to fast bowling. From the findings, in coaching (96.3%, 98.3%) and games (99.6%, 96.9%), the technique was found to be both sophisticated and definite. The study by Anuj Chauhan et al. (56) aimed at eradicating human errors regarding the detection of various activities taking place in cricket by employing technology like computer vision to know the impact of technology on every aspect of life. The study uses techniques of computer vision to detect activities such as critical catches, wide ball, LBW, no ball, and so on. The system imbrutes the varied actions taken during a cricket match, allowing the umpire and coach to make accurate decisions during the game. The study by Arjun Nelikanti et al. (57) focuses on creating a system that helps the cricket on-field umpire make Leg before wicket options including using two cameras located at the on-field umpire's position to note the clip of the batter attempting to play the ball. At first, pre-processing yields data on ball movement. After the batter intercepts the ball a new method known as Spider-Squirrel Optimization-based Deep Long Short-Term Memory (SSO-based deep LSTM) is suggested for route prediction. To examine the leg-before-wicket incident utilizing a forecasting confidence-based judgment, the outcome of the prediction route is taken into consideration. The model's average square error calculation produced the lowest error of 1.107. The imbrute retrieval of significant events and successive overview of sports videos using scoreboard monitoring are proposed in this study by Chakradhar Guntuboina et al. (58) as a low-cost computational method. YOLO (You Only Look Once) a supervised—learning-based object detection algorithm.) is used. The suggested approach is particularly fit for game analysts who require precise dates and times of important events. Yolo obtained 97.1%, 94.4%, and 95.7% for 8 classes in the precision, recall, and f1-score evaluations. The study by Mazhar Javed Awan et al. (59) aims at building a model able to find out the winner of a cricket match determined by entering current game conditions. The model has been employed without big data predictive modelling regression model and big data conceptual model Spark ML to predict the team scores. Using the Spark ML concept, the model has achieved 96% accuracy.

The study by Joseph McGrath et al. (60) aimed to find out if the Inertial measurement unit (IMU) combined with machine learning could estimate two indirect methods of bowling strength: ball discharge speed (BRS) and presumed intensity zone (PIZ). Data was collected by attaching 44 fast bowlers IMUs implanted in their thoracic backs one each. Participants were made to bowl 36 deliveries in each random zone 1 = 24 deliveries at 70%–85% of maximum perceived bowling effort; zone 2 = 12 deliveries at 100% of maximum perceived bowling effort)"4ML techniques were used for analysing Data out of which Gradient boosting models performed consistently and very well Dinithi Hasanika et al. (11) choose Only ODI games Australia, West Indies, Sri Lanka, Bangladesh, New Zealand, Ireland, India, Zimbabwe, Afghanistan, England, South Africa, and Pakistan are full members of the ICC as the subject of this study. The team performances of the players as well as some characteristics unique to the team and the contest are used to predict the outcome. Taking into account all-time ODI data, the individual performances of batsmen, bowlers, and fielders are examined independently. The study used data mining and ML approaches for all of these predictions. The performance analysis and outcome projection together with match data from 2015 to 2020 were taken into consideration. A hybrid machine learning strategy is suggested in this study by Hansa Shingrakhia et al. (61) to summarise cricket videos. It examines elements based on excitement, objects, and events to identify significant moments in the cricket video's-AM is used for Audio analysis, HRF-DBN is used for classifying the sequences of each interesting clip. The SGRNN-AM model is employed to identify significant occurrences, like fours, sixes, and wickets. It has 96.82 percent precision and 96.32 percent accuracy demonstrating its efficacy. The study by Suvarna Nandya et al. (62) initiates with recognizing Umpire postures and organizing events in a cricket match. A new dataset called SNWOLF for the automatic generation of highlights from cricket sport the dataset will be an initial help that has been assessed in system development. The referee's stance from the cricket video referee action frame identified the classification of the most frequently used event: SIX, NO BALL, WIDE, OUT, LEG BYE, and FOUR. Convolutional Neural Networks (CNNs) were used to extract features and classify identified frames into Umpire postures. A completely new dataset of 1,040 images of Umpire Action Images containing these six events was created. The system trains the CNNs classifier on 80% of images of the SNWOLF dataset and tests on 20% of images. The overall accuracy of 98.20% was achieved by the modal average overall accuracy of 98.20% and consolidates on very low cross-entropy loss.

To classify sports videos, this study by M. Ramesh et al. (63) creates an efficient key frame extraction method and a hybrid Wavelet Convolution Neural Network (WCNN) framework with an optimisation scheme. In the beginning, input videos are converted into frames, and keyframes are extracted using the Enhanced threshold with Discrete Wavelet Transform (ETDWT) method. The noise in the keyframe is then removed using the Cross Guided Bilateral Filter (CGBF) method. The Fuzzy Equilibrium Optimizer (FEO) algorithm then performs segmentation, and motions are detected using the Farneback optical flow (OF) method. Finally, to categorise various sports videos, the Hybrid Wavelet Convolutional Manta Ray Foraging Optimisation (HWCMRFO) algorithm is used. Python is used to implement the overall work. The simulation results demonstrated that the proposed work achieved the highest accuracy (93.17%) when compared to others.

## 4. Results and discussion

Using the search results listed above 1,230 articles from the database searches were obtained where a restriction of the English language was imposed. An additional 270 articles were added. After then duplicates were removed resulting in 1,110 results. Then in the reviewing process of titles and abstracts, many results got eliminated and we got 270 full-text articles. Further processing left a total of 40 articles that were chosen for the final sample (Figure 2 is the PRISMA process flow diagram).

### 4.1. Descriptive results

The research papers under review are based on understanding the impact of ICT on cricket as a whole as well as the corresponding domains of cricket. From the literature review, we can see out of 40 studies, 57.5% (23) studies are focussed on impacts on cricket as a whole like a prediction of the outcome of cricket matches, probability of winning, highest scores, etc., 17.5% (7) studies focussed on impacts on batting domain only like classifying bating shots, predicting rising stars in a game, kinematics of the pull shots and much more, 20% (8) studies are focussed on the bowling domain like on delivery detection, predicting the performance of bowlers, etc. and we found 5% (2) studies have taken in consideration wicket keeping domain whereby discussing skills and movements of wicket keepers.

**Figure 3:**
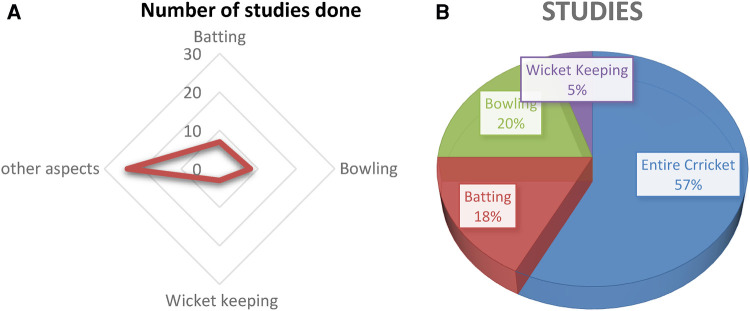
(**A**) Studies that were done in different domains. (**B**) Domain specific distribution.

Figures 3A,B shows the domain distribution of the studies in the cricket sport. Very few (5%) of them deal with wicketkeeping.

**Figure 4:**
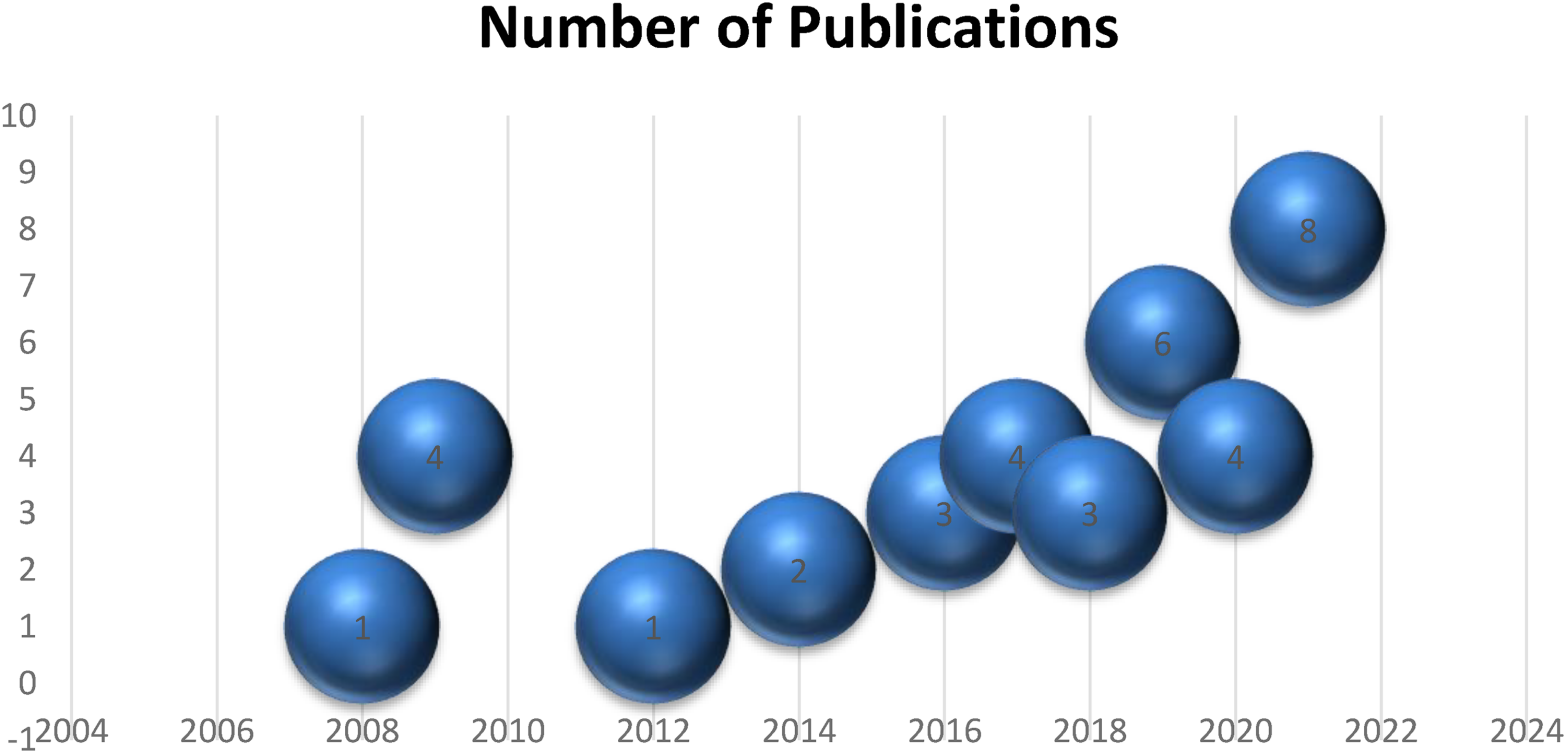
Temporal distribution of research articles.

Figure 4 displays the chronological distribution of articles. The figure shows that interest of the researchers to incorporate technology in sports especially cricket has shown an increase from years 2012 to 2022. With the increasing developments and advances in technology the research interest to incorporate it in sports also increases. However, because the analysis is impartial, the statistics may be interpreted independently of the aim of the survey.

## 5. Conclusion

Technology has revolutionized the world. The modern world is paralyzed if the technological aspect is taken away from it. Technology has its impact on every aspect of life, so like the other aspects, we found the technology to be impacting crucially the sports domain as well. From the review process, we found abundant literature about the use of ICT in sports as a whole as well as in individual sports. The literature gave us a clear vision of how intensely the technology has been introduced in the cricket sport in special. ICT has impacted Cricket sport in various aspects like player performance detection, match outcome prediction, umpire gesture detection, and many more. Altogether, it has impacted the cricket sport vastly. However, another domain of sports talent identification is a bit lagging in this technical aspect. Although many sports have incorporated technology in this aspect as well, however, we found no study for talent identification in cricket employing any modern technology. The review demands a keen interest of researchers in incorporating the technology for talent identification of cricket sport as well as skill-specific talent identification of the performers interested in cricket sport.
