# Supplementary material for: Information communication and technology in sports: a meticulous review
Source: Front Sports Act Living. 2023 Jul 3;5:1199333. doi: 10.3389/fspor.2023.1199333 (PMC10351379; doi:10.3389/fspor.2023.1199333)
Supplement: Supplementary file 1 [file Datasheet1.docx]

Supplementary Material

INFORMATION COMMUNICATION AND TECHNOLOGY IN SPORTS: A METICULOUS REVIEW

Nahida Reyaz^1^, Gulfam Ahamad^2*,^ Mohd Naseem^3,^ and Javed Ali^4^

*** Correspondence:** Gulfam Ahamad: [gulfamahmad@bgsbu.ac.in](mailto:gulfamahmad@bgsbu.ac.in),

# Supplementary Figures and Tables

For our study we have one table and 5 figures as supplementary material.

Although we have changed the tabular literature review into paragraphic form , we still submitted it as a supplementary material into the tabular form.

## Supplementary Table

Table1: Tabular representation of Literature Surveyed.

| Paper Title | | Author | sport/  Domain | Y  e  a  r | | Objectives | Methods | Findings | |
| --- | --- | --- | --- | --- | --- | --- | --- | --- | --- |
| “Prediction of Rising Stars in the Game of Cricket  ” | | [Haseeb Ahmad](https://ieeexplore.ieee.org/author/37085658298) et al,[21] | Cricket | 2017 | The study uses machine learning approaches to find up-and-coming cricket players. More specifically, it foresees rising stars in both the batting and bowling fields. | | Incorporating the ideas of teammates, teams, and rival teams, unique characteristics are provided together with their mathematical formulas. Two models from each category are tested using generative and discriminative machine learning methods for classification purposes. | Study shows rising star prediction with high accuracy that is both reliable and statistically significant using cross-validation. |  |
| “KINEMATICS OF the PULL SHOT OF THE MALAYSIAN NATIONAL CRICKET BATSMEN” | | Muhammad Zia ul Haq et al,[22] | Cricket | 2017 | Despite their best efforts, several cricket specialists could not measure the mechanics of the motion of the pull shot technique. The concerned research compared the pull shot techniques of under-19, under-16, and senior cricket batsmen to quantify coaching descriptions. | | The pull shot action was recorded using two high-speed video cameras, and the kinematics analysis was performed using Aerial Performance Analysis Software. To compare the biomechanics of the pull shot between groups, a one-way ANOVA with repeated measures was used. | As per the study, senior batsmen extended their right knee and left hip much more than batsmen under 16 and batsmen under 19. The left hip's linear velocity was much higher in the under-16 batsmen than it was in the seniors, and the left elbow was higher in the under-19 hitters. |  |
| “Deep CNN Based Data-Driven Recognition of Cricket Batting” | | [Muhammad Zeeshan Khan](https://ieeexplore.ieee.org/author/37086517258) et al,[23] | Cricket | 2018 | This study looks at several batting shots from cricket films and classifies them. Deep convolution neural networks are used, and it highlights the significant indications of contemporary AI and deep learning for both identifying different cricket activities and for decision-making. | | Both 2D convolution and recurrent networks were used to analyze a sequence of video frames, while 3D convolution networks were used to concurrently capture spatial and temporal data. | 90% of the time, obtained models can accurately identify a shot being played. The ability to distinguish between two strokes that are visually identical with such high accuracy is unprecedented in literature |  |
| “Intelligent Cricket Team Selection by Predicting Individual Players’ Performance using Efficient Machine Learning Technique” | | Chetan Kapadiya  et al,[24] | Cricket/ Artificial intelligence | 2020 | The study suggests a model for predicting the Players' effective performance in the cricket game. The presented model refines entire team effectiveness helping in the best team selection. | | Cricket statistics, as well as weather-related data datasets are utilized by the model. Then the pre-processing has been done to balance the dataset. The model employs a weighted random forest classifier with optimisation. The model is then tested on the balanced dataset. | The accuracy of the predicted model is found to be good as compared to other algorithms. |  |
| “”Cric Shot Classify: An Approach to Classifying Batting Shots from Cricket Videos Using a Convolutional Neural Network and Gated Recurrent Unit” | | [Anik Sen](https://pubmed.ncbi.nlm.nih.gov/?term=Sen%20A%5BAuthor%5D) et al,[25] | Cricket | 2021 | To categorize batting shots of 10 types from offline footage, this research suggests a mixed deep-neural network design. The CricShot10 dataset was created by the study and contains batting shots of various lengths and variable lighting. | | An automatic convolutional neural network (CNN), is a system that handles feature engineering., while long temporal dependencies are handled by a gated recurrent unit (GRU). Initially, CNN-based architectures that were both conventional and dilated were constructed. Then, various transfer-learning models that freeze all the layers—namely, VGG16, InceptionV3, Xception, and DenseNet169—were examined. | Results of the experiment showed that the VGG16-GRU model performed better than outperformed the other models with an accuracy of 86%. |  |
| “Data Analytics in the Game of Cricket: A Novel Paradigm  ” | | [VaradVishwarupe](https://www.sciencedirect.com/science/article/pii/S1877050922008523#!) et al,[26] | Cricket | 2022 | Based on a case study and real-world IoT implementation, the study sought to identify a fresh paradigm of research in cricket analytics known as the timing index. The timing index is dependent on a variety of variables, including impact bat speed, maximum bat speed, back lift angle, and bat speed. | | The cricket bat sensor can be attached to the top or back of the bat, and it uses numerous integrated sensors to record both the player's motion and the movement of the bat during the shot. We employed the aforementioned Stance Beam cricket bat sensor for this study. A sensor of around 5 cm in size is attached to the apex of the cricket bat in the Stance Beam cricket bat sensor. | Such a study is thought to be able to analyze a player's practice sessions utilizing IoT and data analytics, accurately and comprehensively analyzing a player's performance, and going beyond conventional statistical reporting by introducing fascinating and illuminating information. |  |
|  |  |  |  |  |  |  |  |  |  |
| Paper Title | | Author | Sport/domain | Year | Objectives | | Methods | Findings |  |
|  |  |  |  |  |  |  |  |  |  |
| “Bowler Performance Prediction for One-Day InternationlCricket Using Neural Networks” | | Shanthi Muthuswamy et al,[28] | Cricket/CNN | 2008 | A neural network technique utilizing a back-propagation algorithm network (BPN) and its radial basis function network (RBFN) has been explored to estimate the effectiveness of the bowlers on the Indian cricket team. Prediction models performed better for runs than for wickets. | | The two network paradigms mentioned were used to create a classification strategy for the wickets case. The effectiveness of the BPN and RBFN concepts was evaluated for the forecasting and classifying scenario. | For runs as compared to wickets, prediction models were more successful. |  |
| “Variability in Movement Patterns During One Day Internationals by a Cricket Fast Bowler” | | Carl Petersen et al,[29] | Cricket/GPS | 2009 | The study aimed at the quantification for the same male fast bowler playing One Day International (ODI) cricket matches, time-motion characteristics and intra-athlete diversity in motion patterns were studied. | | For monitoring several different time-motion characteristics GPS (Global Positioning System) portable 5-Hz unit (Catapult, Melbourne, Australia) was used. | It was found that a combination of high-intensity, intermittent activities on a 16-kilometer basis created the manner in which this fast bowler moves. Further, adapting the learning and retrieval procedures of ODI fast bowlers offer overview for adjusting coaches in determining their physical requirements. |  |
| “Analysis of Performance of Bowlers using Combined Bowling Rate” | | [Dibyojyoti Bhattacharjee](https://www.semanticscholar.org/author/Dibyojyoti-Bhattacharjee/3369101) et al ,[30] | Cricket  /AI | 2012 | This study makes an effort to evaluate the effectiveness of a data set of bowlers who participated in the season 4 of the Indian Premier League using the combined bowling rate. | | The reasons that are empirically accountable for the bowlers' performance were then determined using multiple linear regression techniques. | Variation in ball speed has a negligible impact on how well a bowler performs.  With a corresponding the variance ratio between the regression equation and the residuals is given as 3.394 with a p-value of 0.015. |  |
| “Quantifying individual performance in Cricket — A network analysis of batsmen and bowlers  ” | | Satyam Mukherjee et al,[31] | Cricket | 2014 | Social network analysis (SNA) is used in the study to analyze the effectiveness of a team's players. | | The study creates a weighted and directed network of batsmen-bowlers using player-vs-player data from Test and ODI cricket. | Our findings indicate that M. Muralitharan is the most productive bowler in cricket history. |  |
| “Predicting the Performance of Bowlers in IPL: An Application of Artificial Neural Network” | | [Hemanta Saikia](https://www.tandfonline.com/author/Saikia%2C+Hemanta)  et al,[32] | Cricket/AI | 2017 | The research purpose is to use an artificial neural network to examine and forecast how bowlers will perform in the IPL. | | Based on how they performed in the league's first three seasons, the paper attempts to forecast the results of bowler who decided to join the league in its season 4 as their initial IPL venture. | Player real performance in IPL-IV is used to assess the model's external validity. |  |
| “ A CNN-based approach to classifying cricket bowlers based on their bowling actions” | | [M. N. A. Islam](https://www.semanticscholar.org/author/M.-N.-A.-Islam/152992964) et al ,[33] | Cricket/CNN | 2019 | This research suggests a CNN model that uses transfer learning to categorize 18 various cricket bowlers based on their bowling style movements. For training the suggested framework and assessing its effectiveness, a brand-new dataset of 8100 photographs of these 18 bowlers was also produced. | | For the model, the study started with the VGG16 model that had already been trained using the ImageNet dataset. | The model's test set precision is 93.3%, demonstrating its classification performance. |  |
| “Effect of different bowling surfaces on bowling speed of pace bowlers: A cross-sectional s  study” | | Amrinder Singh et al,[34] | Cricket | 2021 | Bowling speed is an important aspect that can affect the outcome of any match, so sports professionals are constantly drawn to it to research and analyze it in depth. | | The purpose of this study was to investigate the effect of different bowling surfaces (natural turf and concrete) on the bowling speed of 41 asymptomatic fast and medium-pacer bowlers who had not sustained any injuries in the three months before they participated in the study, particularly to the shoulder joint or back. This one-time cross-sectional design investigation included measurements of bowling velocity and physical profile using an SRA 3000 Tracer Precision Radar gun. | This study examines the statistical analysis of the observational data using the average value, standard error, and t-value and concludes that throwing on a cement-based pitch reduces ball speed slower than pitching on natural turf. The statistics indicate a decrease in pitch tempo, which is the difference in pitch speed between the two ends of the pitch, on a pitch with a concrete foundation. |  |
| “Deep Grip: Cricket Bowling Delivery Detection with Superior CNN Architectures  ” | |  |  | 2021 | In this paper, a unique method for determining the delivery style from a bowler's finger grip during delivery was proposed. The primary goal of this research is to accurately classify bowlers' grips using the transfer learning models and the prototype CNN architecture. | | To train with the GRIP DATASET and analyse grip results, as well as the pre-trained transfer learning models Vgg16, Vgg19, ResNet101, ResNet52, DenseNet, MobileNet, AlexNet, Inception V3, and NasNet - were used. | It was tested for precision, recall, and f1-score, with a maximum average accuracy of 98.6% achieved across 13 classes. (13 different bowling actions). |  |
|  |  |  |  |  |  |  |  |  |  |
|  |  |  |  |  |  |  |  |  |  |
| Paper Title | | Author | Sport/domain | Year | Objectives | | Methods | Findings |  |
| “Quantifying positional movement patterns in Twenty20 cricket” | | Petersen et al,[37] | Cricket/ GPS | 2009 | The study quantified the time-motion characteristics of five cricket positions ( Batters, Fast bowlers, Position players, Spin bowlers, and Wicketkeepers) competed in four State Twenty20 (T20) cricket games. | | The study used portable 5Hz global positioning system (GPS) units | The study found that the physical demands of Fielders and Fast bowlers are substantially greater than wicketkeepers and spin bowlers |  |
| “Key movements and skills of wicketkeepers in one-day international cricket” | | Dani MacDonald et al[38] | Cricket/computer vision | 2018 | The study aimed to find out the skill demands and movement in the one-day international match by the use of video analysis. | | Using the video analysis program Sports Code, television footage from eight games (totalling sixteen innings) of the 2011 one-day international World Cup was examined. | The study's findings can help practitioners better grasp the wicket-keeper position's assessment, program, and skill development components. |  |
|  |  |  |  |  |  |  |  |  |  |
| “Prediction of athlete’s performance using neural networks: An application in cricket team selection  ” | | [Subramanian RamaIyer](https://www.sciencedirect.com/science/article/abs/pii/S095741740800420X#!) et al[39] | Cricket  /AI | 2009 | The study employs neural networks to forecast how well each cricketer will perform in the future based on their previous performance. Cricket players are classified as either performers, middling performers, or failures. | | The neural network models were gradually trained and tested using four sets of data. The trained neural network models were then used to estimate the cricketer's relatively close performance. | The model has a 77% accuracy rate for batting performance and a 63% accuracy rate for bowling performance. |  |
| “Quantifying positional movement patterns in Twenty20 cricket” | | Petersen et al,[37] | Cricket/ GPS | 2009 | The study quantified the time-motion characteristics of five cricket positions (Batsmen, Fast bowlers, Fielders, Spin bowlers, and Wicketkeepers) in four State Twenty20 (T20) cricket matches. | | The study used portable 5Hz global positioning system (GPS) units | The study found that the physical demands of Fielders and Fast bowlers are substantially greater than wicketkeepers and spin bowlers |  |
| “Auto-play: A Data Mining Approach to ODI Cricket Simulation and Prediction  ” | | [Vignesh Veppur Sankaranarayanan](https://epubs.siam.org/author/Sankaranarayanan%2C+Vignesh+Veppur), et al [40] | Cricket/Data  mining | 2014 | To forecast future match events that would result in a win or lose, research developed a prediction system that takes into account both data from previous matches as well as the degree of development of a match. | | Using a selection of match parameters and a hybrid of nearest-neighbour clustering and linear regression, the study models the game. | Quantitative findings showed that one of the key factors affecting match outcome is how well our algorithms forecast the number of runs scored. |  |
| “Application of computer vision in Cricket: Foot oversteps no-ball detection  ” | | [A Z M Ehtesham Chowdhury](https://ieeexplore.ieee.org/author/37085697431) et al,[41] | Cricket/Computer vision | 2016 | The study aimed at concretizing the decision regarding the occurrence or non-occurrence of the no ball. | | The study made two divisions of the bowling crease, the transformation in pixels then was calculated using the picture decrement technique on two areas. | The study eradicated the inadequate nature of human perception as it is based on a pixel-by-pixel image subtraction. |  |
| “Applications of modern classification techniques to predict the outcome of ODI Cricket” | | Neeraj Pathak  et al,[42] | Cricket | 2016 | This study aims to forecast the result of a One Day International (ODI) cricket match. The match's outcome is influenced by several variables, many of which change as the game progresses, including home field advantage, Day/Night, Toss, Innings (first or second), physical fitness of sides, and dynamic plans. | | The study compares the results and performances of three contemporary classification techniques—Naive Bayesian, Support Vector Machines, and Random Forest. COP (Cricket Outcome Predictor), a tool that predicts the outcome of an ODI match, was developed with reference to the outcomes of these models. | Average balanced accuracy of models:  Random Forest 0.6002, SVM 0.6167, and Nave Bayesian 0.6018 |  |
| “Predicting the Outcome of ODI Cricket Matches: A Team Composition Based Approach” | | Madan Gopal  et al,[43] | Cricket/ | 2016 | This article uses supervised learning to attempt to forecast the outcome of a One Day International (ODI) cricket match based on the team composition. The research reveals that the relative team strength of the competing teams is a distinguishing feature for determining the winner. | | The player was modelled in the study using both his current performances and career statistics. Player-independent elements have also been considered in order to forecast the outcome of a game. | Based on the statistics of 366 matches, the study's accuracy was 71%. |  |
| “Activity Recognition for quality Assessment of Bating Shots in Cricket using a Hierarchical Representation” | | AFTAB KHAN et al,[44] | Cricket/ | 2017 | The study offers a framework for the automated recognition of cricket shots that is both affordable and practical. The movements of the batsmen are captured using body-worn inertial measurement units, and the data is subsequently analyzed using a parallelized, hierarchical recognition system that automatically identifies pertinent categories of strokes as necessary for evaluating batting quality. | | The technology produces accurate visual representations of important performance indicators, such as foot placement, attack/defense, and shot distribution on the playing surface. These visualizations serve as the foundation for an objective skill assessment, concentrating on particular areas for individual growth that the system has identified. | F1-score greater than 88% |  |
| “Automatic Cricket Highlight Generation Using Event-Driven and Excitement-Based Features  ” | | [Pushkar Shukla](https://ieeexplore.ieee.org/author/37085640522)  et al[45] | Cricket/Artificial Intelligence | 2018 | The study suggests a model that can create sports highlights automatically, with a concentration on cricket. | | This research proposes a strategy to identify and clip significant occurrences, the system imbrutes the varied actions taken during a cricket match, allowing the umpire and coach to make accurate decisions during the game. excitement-based attributes. Examples of Replays, audio intensity, player celebration, and playfield scenarios are all used to record such events. CNN + SVM. | 72.31% is average precision. |  |
| “A Dataset and Preliminary Results for Umpire Pose Detection Using SVM Classification of Deep Features” | | [Aravind Ravi](https://ieeexplore.ieee.org/author/37086611087)  et al,[46] | Cricket/AI | 2018 | To detect umpire poses in the game of cricket, the study proposes a novel dataset named SNOW. The proposed dataset is assessed as a first step in creating cricket highlights-generating software. | | According to the study, four such events—SIX, NO BALL, OUT, and WIDE—can be classified based on the umpire's pose as seen in cricket video frames. As the top contenders for feature extraction prior to training convolutional neural networks such as the Inception V3 and VGG19 networks are used. | Player testing accuracy for VGG19-Fc2 was 78.21%. |  |
| “A Multimodal Approach for Automatic Cricket Video Summarization.”  ” | | [Aman Bhalla](https://ieeexplore.ieee.org/author/37088534880) et al,[47] | Cricket/ Computer vision | 2019 | The study suggests for cricket matches a unique strategy for instantly summarising and designed to detect significant events. | | The input to the model is the entire cricket match in a form of video recorded, and the most important clips from the game are output. Many approaches have been used such as sound detection, optical character recognition, and replay detection to excerpt crucial events like wickets, boundaries, and other playfield schemes. | The model achieved an 89.45% accuracy in detecting events like wickets, fours, and sixes indicating the significance of the designed technique. |  |
| “Detecting Third Umpire Decisions & Automated Scoring System of Cricket  ” | | [Md. Kowsher](https://ieeexplore.ieee.org/author/37087125788)  et at,[48] | Cricket / Computer vision | 2019 | The study presents a classification method Convolutional Neural Network (CNN) with Inception-V3 so that decisions of the third umpire and scoring system can be automatically unravelled like signal detection. | | SoftMax has been applied to find out the likelihood of a match referee judgement and match referee signal classification.  In order to train CNN, pre-formed-V3. was used. | The efficacy and effectiveness of the model are found to be high as compared to other methods and applications for detecting no-balls. |  |
| “Shot-Net: A Convolutional Neural Network for Classifying Different Cricket Shots” | | [Md. Ferdouse Ahmed Foysa](https://link.springer.com/chapter/10.1007/978-981-13-9181-1_10#auth-Md__Ferdouse_Ahmed-Foysal) et al,[49] | Cricket/ Artificial intelligence | 2019 | A 13-layered CNN known as "Shot-Net" is used. suggested by the study to classify six different categories of cricket shots played viz. Pull Shot, Scoop Shot, Cut Shot, Cover Drive, Straight Drive, and Leg Glance Shot. | | The study has focussed on employing Deep Neural Networks as being very useful in different sports data analysing tasks. | The accuracy achieved by the model is fairly high and also entropy is low. |  |
| “Outcome Classification in Cricket Using Deep Learning  ” | | [Rohit Kumar](https://ieeexplore.ieee.org/author/37088356680)  et al,[50] | Cricket/ Artificial intelligence | 2019 | In this study, cricket films were used for the outcome categorization job. The creation of automatic commentary generation is the major goal of such actions. For this assignment, there are a lot of sub-tasks that must be taken into account. Classifying the result of each ball for which commentary is to be produced is one of those duties. | | This paper covers the entire categorization process, from gathering the data to producing the findings. Using Long Short-Term Memory with Convolutional Neural Networks in the game of cricket, there are four main outcomes: Run, Dot, Boundary, and Wicket. | Cricket match ball-by-ball videos' results have been predicted with an accuracy of 70%. |  |
| “The Cricket Winner Prediction with Application of Machine Learning and Data Analytics” | | Daniel Mago Vistro et al,[51] | Cricket/ Artificial intelligence | 2019 | The study aims at predicting the IPL match winner prior to the game starts. To predict the winner of the IPL machine learning models is trained on the designated features. | | Different advanced machine learning algorithms are put in for the reason of model building on various test and training datasets like Logistic Regression, Random Forest, SVM, Decision Tree, and Naïve Bayes. | For evaluating the team’s strength and cricket analysis the model is found to perform well. |  |
| “Auto detecting deliveries in elite cricket fast bowlers using microsensors and machine learning” | | [Hannah K Jowitt](https://www.tandfonline.com/author/Jowitt%2C+Hannah+K)  et al,[52] | Cricket/ Artificial intelligence | 2020 | The study for detecting bowling deliveries accurately, automatically, and reliably aimed to devise and check the logic based on a machine learning approach. | | Inertial sensor data from a Catapult OptimEye S5 wearable device was collected from national as well as global grade fast bowlers (n = 35) in both practice and competition at various intensities. A machine-learning-based algorithm comes out to be a reliable tool for detecting bowling events instantaneously, moreover, enabling us to look at performance metrics related to fast bowling. | From the findings, in coaching (96.3%, 98.3%) and games (99.6%, 96.9%), the technique was found to be both sophisticated and definite. |  |
| “Cricket Activity Detection Using Computer Vision” | | Anuj Chauhan et al,[53] | Cricket / Computer vision | 2020 | The study aimed at eradicating human errors regarding the detection of various activities taking place in cricket by employing technology like computer vision to know the impact of technology on every aspect of life. | | The study uses techniques of computer vision to detect activities such as critical catches, wide ball, LBW, no ball, and so on. | The system imbrutes the varied actions taken during a cricket match, allowing the umpire and coach to make accurate decisions during the game. |  |
| “An Optimization-Based deep LSTM Predictive Analysis for Decision Making in Cricket” | | [Arjun Nelikanti](https://www.researchgate.net/scientific-contributions/Arjun-Nelikanti-2189337003?_sg%5B0%5D=bHB2zWWkOybGhIl_5V3XTg_L0pMJwWSVOogFrCaH71pEWjl6qg-swVcvFqqHbOC6Zj3GF0k.Bykcsx33ZH-cBUfmS1mKsBIizK7pHrIj8G9zX6iUJsgqdyJI6UzuZ8-TYwAwfUbQuuphgBmDAcpSafQR5Qp6hw&_sg%5B1%5D=mKtF5qvcI-Ryigq5ZVYDjR3xCo_AsziBqPlMiap69XAHYXFelcapadIneA-MBmRnCymNTwU.f6c8CnGsAM93EmdbD_uLYOOKToo10cFl3Lws5k11kwrftYzUYTPrUckL28itaWURsw8ZJ_1hERPdJxZbBPwn-A) et al,[54] | Cricket | 2021 | This study focuses on creating a system that helps the cricket on-field umpire make Leg before wicket options include using two cameras located at the on-field umpire's position to note clip of the batter attempting to play the ball. | | At first, pre-processing yields data on ball movement. After the batter intercepts the ball a new method known as Spider-Squirrel Optimization-based Deep Long Short-Term Memory (SSO-based deep LSTM) is suggested for route prediction. To examine the leg-before-wicket incident utilising a forecasting confidence-based judgment, the outcome of the prediction route is taken into consideration. | The model's average square error calculation produced a lowest error of 1.107. |  |
| “Deep Learning Based Automated Sports Video Summarization Using Yolo” | | Chakradhar Guntuboina et al,[55] | Cricket | 2021 | The imbrute retrieval of significant events and successive overview of sports videos using scoreboard monitoring are proposed in this study as a low-cost computational method. | | YOLO (You Only Look Once) a supervised - learning-based object detection algorithm.) is used. The suggested approach is particularly fit for game analysts who require precise dates and times of important events. | Yolo obtained 97.1%, 94.4%, and 95.7% for 8 classes in the precision, recall, and f1-score evaluations. |  |
| “Cricket Match Analytics Using the Big Data Approach  ” | | [Mazhar Javed Awan](https://sciprofiles.com/profile/779111) et al,[56] | Cricket/ Artificial intelligence | 2021 | The study aims at building a model able to find out the winner of a cricket match determined by entering current game conditions. | | The model has employed without big data predictive modelling regression model and big data conceptual model Spark ML to predict the team scores. | Using the Spark ML concept, the model has achieved 96% accuracy. |  |
| “Can an inertial measurement unit (IMU) in combination with machine learning measure fast bowling speed and perceived intensity in cricket?  ” | | [Joseph McGrath](https://www.tandfonline.com/author/McGrath%2C+Joseph) et al,[57] | Cricket/ Artificial intelligence | 2021 | The study aimed to find out if Inertial measurement unit (IMU) combined with machine learning could estimate two indirect methods of bowling strength: ball discharge speed (BRS) and presumed intensity zone (PIZ). | | Data was collected by attaching 44 fast bowlers IMUs implanted in their thoracic backs one each. Participants were made to bowl 36 deliveries each randomly zone 1 = 24 deliveries at 70% to 85% of maximum perceived bowling effort; zone 2 = 12 deliveries at 100% of maximum perceived bowling effort)" | 4 ML techniques were used for analyzed Data out of which Gradient boosting models performed consistently and very well. |  |
| “Data Mining System for Predicting a Winning Cricket Team” | | Dinithi Hasanika et al,[9] | Cricket/  Data mining | 2021 | Only ODI games Australia, West Indies, Sri Lanka, Bangladesh, New Zealand, Ireland, India, Zimbabwe, Afghanistan, England, South Africa, and Pakistan are full members of the ICC. are the subject of this study. | | The team performances of the players as well as some characteristics unique to the team and the contest are used to predict the outcome. Taking into account all-time ODI data, the individual performances of batsmen, bowlers, and fielders are examined independently. The study used data mining and ML approaches for all of these predictions. | The performance analysis and outcome projection together with match data from 2015 to 2020 were taken into consideration. |  |
| “SGRNN-AM and HRF-DBN: a hybrid machine learning model for cricket video summarization” | | [Hansa Shingrakhia](https://www.researchgate.net/profile/Hansa-Shingrakhia?utm_content=businessCard&utm_source=publicationDetail&rgutm_meta1=AC%3A4103112) et al,[58] | Cricket/AI | 2022 | A hybrid machine learning strategy is suggested in this study to summarise cricket videos. It examines elements based on excitement, objects, and events to identify significant moments in the cricket video. | | SGRNN-AM is used for  Audio analysis, HRF-DBN is used for classifying the sequences of each interesting clip. The SGRNN-AM model is employed to identify significant occurrences, like fours, sixes, and wickets. | It has 96.82 percent precision and 96.32 percent accuracy demonstrating its efficacy. |  |
| “Cricket Event Recognition and Classification from Umpire Action Gestures using Convolutional Neural Network” | | Suvarna Nandya  et al,[59] | Cricket/Artificial intelligence | 2022 | The study initiates with recognizing Umpire postures and organizing events in a cricket match. A new dataset called SNWOLF for the automatic generation of highlights from cricket sport the dataset will be an initial help that has been assessed in system development. The referee's stance from the cricket video referee action frame identified the classification of the most frequently used event: SIX, NO BALL, WIDE, OUT, LEG BYE, and FOUR. | | Convolutional Neural Networks (CNNs) were used to extract features and classify identified frames into Umpire postures. A completely new dataset of 1040 images of Umpire Action Images containing these six events was created. The system trains the CNNs classifier on 80% of images of the SNWOLF dataset and tests on 20% of images. | The overall accuracy of 98.20% was achieved by the modal average overall accuracy of 98.20% and consolidates on very low cross-entropy losses. |  |

## Supplementary Figure 1.

##
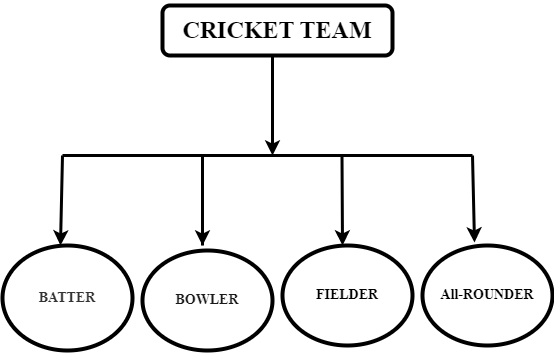


Figure1: Categories of players in cricket teams based on roles played by them.

## Supplementary Figure 2.


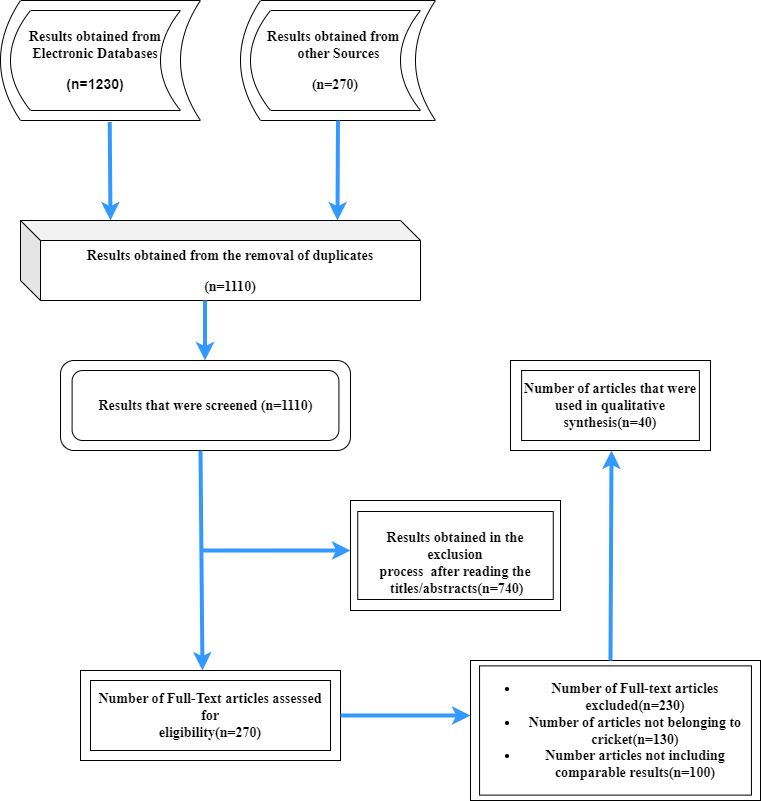


**Figure 2**: Screening Process of Articles for Review

## Supplementary Figure3.

Figure 3(A): Studies done in different domains.

Figure 3(B): Domain specific study.

## Supplementary Figure4.

Figure 4: Temporal distribution of research articles.
